# Environmental Risk and Adverse Perinatal Health Indicators in New York City: A Geospatial Hotspot Analysis

**DOI:** 10.1007/s11524-026-01060-8

**Published:** 2026-03-20

**Authors:** Alexis R. Grayon, Linda G. Kahn, Leonardo Trasande, David C. Lee, Carol Duh-Leong

**Affiliations:** 1 Department of Pediatrics, New York University Grossman School of Medicine, New York, NY USA; 2 Department of Population Health, New York University Grossman School of Medicine, New York, NY USA; 3 Ronald O. Perelman Department of Emergency Medicine, NYU Grossman School of Medicine, New York, NY USA

**Keywords:** Environmental exposure, Perinatal health, Geospatial

## Abstract

Environmental exposures can have adverse associations with perinatal health and birth outcomes. This study aimed to identify the overlap and association between urban areas of environmental risk and adverse perinatal health indicator hotspots in New York City. We examined 2101 census tracts representing 575,257 births from 2016 to 2020 recorded by the New York City Bureau of Vital Statistics looking at preterm birth, adolescent pregnancy, and pre-pregnancy obesity rates. The Getis-Ord Gi* statistic was used to identify geospatial hotspots of adverse indicators. We used multivariable logistic regression to assess associations between areas of environmental risk and odds of indicator hotspot status and Poisson regression to assess associations of hotspot overlap. Overall, 54.6% of environmental risk areas were hotspots for at least one adverse perinatal indicator, accounting for 63.7% of preterm birth hotspots, 93.7% of adolescent pregnancy hotspots, and 67.3% of pre-pregnancy obesity hotspots. Compared with non-risk areas, risk areas had greater odds of being a hotspot of preterm birth (aOR = 2.11; 95% CI 1.60–2.78), adolescent pregnancy (aOR = 32.8; 21.8–49.4), and pre-pregnancy obesity (aOR = 3.15; 2.56–3.87). Environmental risk areas were expected to have 3.36 times the number of overlapping hotspots after adjusting for parental birthplace and parity. The overlap between environmental risk areas and hotspots of adverse perinatal health indicators and the associations with individual indicators and overlapping hotspots suggest that environmental risk area designation may be a useful measure of perinatal health vulnerability for targeted community interventions.

**Supplementary Information:**

The online version contains supplementary material available at 10.1007/s11524-026-01060-8.

## Introduction

**Fig. 1:**
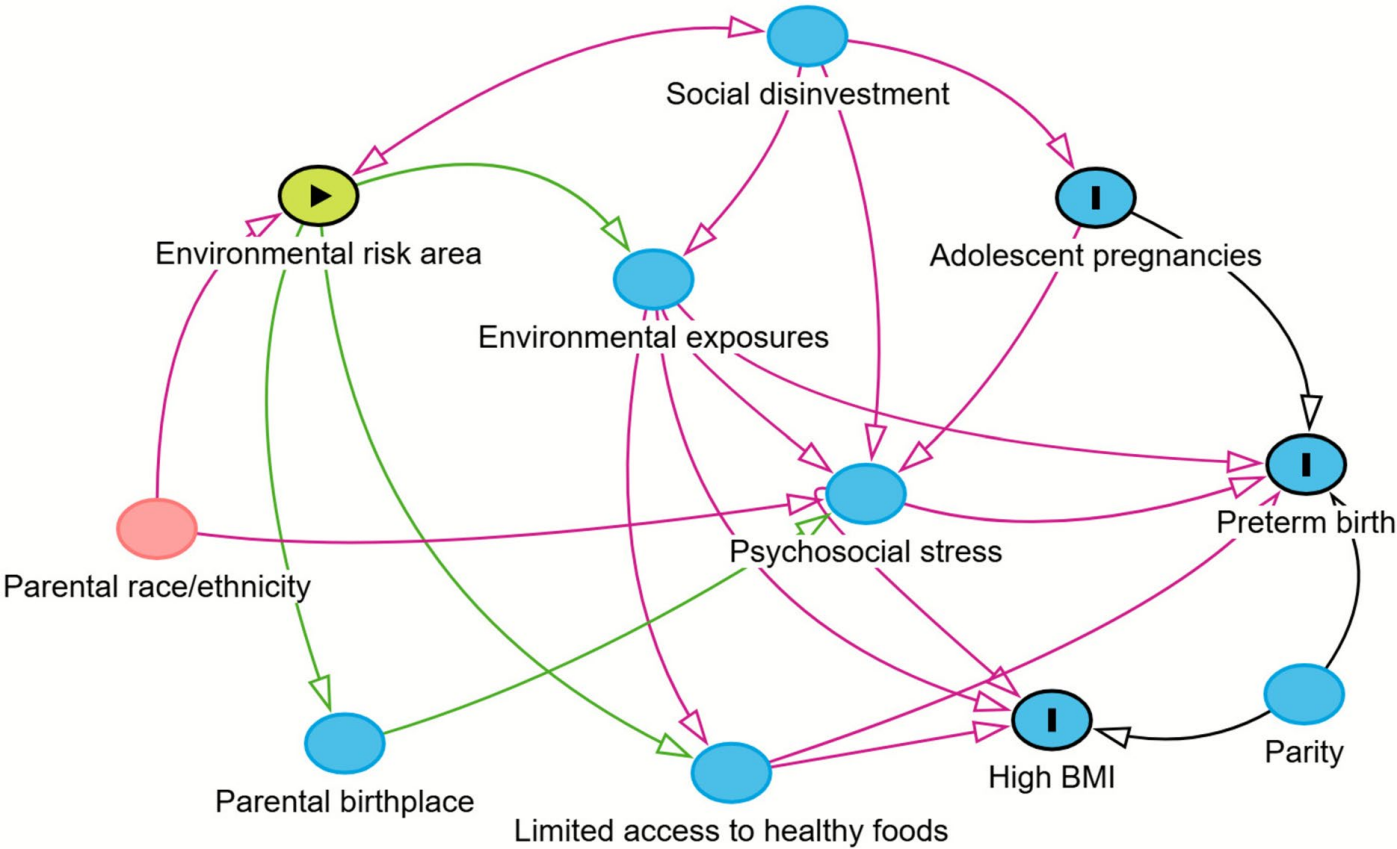
Directed acyclic graphs for variables of interest

Protecting perinatal health from environmental risks is a public health priority to safeguard the well-being of current and future generations. Perinatal health indicators include maternal and birth-related measures closely monitored by health departments such as preterm birth, adolescent pregnancies, and pre-pregnancy obesity status [1]. Growing evidence shows that perinatal health indicators are likely shaped by external environmental conditions surrounding pregnancy [2, 3]. Researchers have observed associations between environmental risk measures and conditions that contribute to adverse perinatal indicators such as hypertensive disease, placenta-related complications, and compromised immune function [4]. Studies have traditionally analyzed these indicators at the individual level, but emerging evidence from geographic studies at county and national levels suggests that occurrences of adverse perinatal indicators cluster together or exhibit spatial proximity [5–7]. Assessing geographic areas of multiple adverse perinatal indicators together and their potential overlap with regions of environmental risks (Fig. 1) would enhance our understanding of where drivers of differences in perinatal health are concentrated and inform strategies to address them.

Policymakers have begun to integrate cumulative sources of environmental exposures guided by the lived experience of affected communities [8, 9]. In New York City (NYC), Local Laws 60 and 64 were established in 2017 to mitigate differences in environmental exposures through the fair and meaningful involvement of people from all backgrounds in creating and enforcing policies that provide universal and balanced access to a healthy environment. They have identified areas of environmental risk within NYC to signify areas disproportionately burdened by environmental hazards [10]. There is a limited understanding of whether these integrated measures overlap with or are linked to areas of heightened adverse perinatal indicators. Examining these indicators together may reveal different patterns that are more likely to arise directly through exposure to environmental exposures (e.g., preterm birth) or indirectly through persistent poverty and lack of opportunity (e.g., adolescent pregnancy), providing valuable insights into how public health resources can be strategically allocated to address these challenges.

To bridge these knowledge gaps and enhance our ability to understand underlying mechanisms and implement solutions, we examined relations between policy-defined areas of environmental risk and geographic hotspots of adverse perinatal indicators. We conducted a geospatial analysis of NYC census tracts to identify geographic hotspots of rates of preterm births, adolescent pregnancies, and pre-pregnancy obesity. We hypothesized that hotspots of adverse perinatal health would overlap with environmental risk areas and that risk area designation would predict hotspots of adverse perinatal health indicators.

## Methods

### Study Design

This was an observational geospatial analysis of publicly available data from the NYC Bureau of Vital Statistics on perinatal health indicators. Our target population included all live births in New York City between 2016 and 2020. Our unit of analysis was a census tract, a geographic unit of measure drawn by the United States Census Bureau that represents about 4000 people [11]. We assessed the distribution of hotspots—defined as statistically significant geospatial clusters—of rates of preterm birth, adolescent pregnancy, and pre-pregnancy obesity. We assessed their overlap and associations with environmental risk area designation. These data are all from publicly available and deidentified databases. The NYU Grossman School of Medicine institutional review board guidelines deemed this study non-human subjects’ research.

### Adverse Perinatal Health Indicators

The NYC Bureau of Vital Statistics records all live births occurring within the city, including those to NYC residents and non-residents who experience this vital event within the city limits. These data are reported by census tract as aggregated 5-year counts. We chose to use the 2016–2020 data, [12] as it is the most recently published tract-level birth dataset, representing 575,257 births.

For each census tract, we divided the occurrence of each adverse perinatal indicator by the total number of newborns per census tract. We selected three available perinatal indicators based on their importance to public health: (1) *preterm birth*, defined as an infant born earlier than 37 weeks of gestation, which increases the risk of various health complications in offspring including respiratory and behavioral problems [13]; (2) *adolescent pregnancy*, defined as a parent who gave birth under the age of 19 years old, which is a marker of maternal and infant health, reproductive healthcare access, and broader social and economic privations [14]; and (3) *pre-pregnancy obesity*, defined as a pre-pregnancy body mass index ≥ 30 kg/m^2^, which has been associated with suboptimal fetal growth and childhood obesity risk [15].

### Policy-Defined Areas of Environmental Risk

The NYC Mayor’s Office classified certain census tracts as areas of environmental risk based on a total of 45 sociodemographic and environmental indicators collected by a collaborative Working Group (Table 1). This group compiled census tract-level data between 2010 and 2021 to calculate percentile ranks, weighted factor scores, and component scores, which were combined into a single score for each tract. All data collected after 2020 represents indicators related to historical infrastructure within each tract. They established a cutoff based on statewide and regional scores, subsequently designating areas that exceeded this threshold as high risk [16]. This geospatial measure is unique because it provides a comprehensive assessment of environmental vulnerability including environmental hazards data rather than focusing solely on overall social circumstances.

**Table 1:** Indicators included in environmental risk area designation

Components	Factor	Indicators
Environmental burdens and climate change risk	Potential pollution exposures	Particulate matter (PM2.5) air concentration
		Benzene air concentration
		Proximity to wastewater discharge
		Diesel trucks and bus traffic
		Vehicle traffic density
	Land use and facilities associated with historical discrimination or disinvestment	Industrial, mining, and manufacturing land use
		Agricultural land use
		Proximity to remediation sites
		Proximity to risk management plan sites
		Proximity to major oil storage facilities
		Proximity to power generation facilities
		Proximity to active landfills
		Proximity to municipal waste combustors
		Proximity to scrap metal processing and vehicle dismantlers
		Housing vacancy rates
	Potential climate change risks	Flooding in coastal and tidally influenced areas (projected)
		Flooring in inland areas (projected)
		Projected days above 90° Fahrenheit
		Low vegetative cover
		Driving time to hospitals or urgent/critical care
Population characteristics and health vulnerabilities	Income, education, and employment	Population earning < 80% of area median income
		Poverty rate (< 100% federal poverty line)
		Single parent households
		Adults without a bachelor’s degree
		Unemployment rate
	Race, ethnicity, and language	Black or African American population
		Hispanic and Latino population
		Asian and Asian American population
		Native American or Indigenous population
		Limited English proficiency
		Historical redlining
	Health outcomes and healthcare	Asthma emergency department visits
		COPD emergency department visits
		Myocardial infarction hospitalizations
		Premature deaths
		Low birth weight births
		Population with a disability
		Population over age 65
		Percentage without health insurance
	Housing mobility and communication	Rented housing units
		Rental housing cost burden
		Energy affordability
		Manufactured and mobile homes
		Households without internet access
		Homes built before 1960

### Statistical Analysis

We identified hotspots of adverse perinatal indicators using the Getis-Ord Gi* statistic to show statistically significant geographic clusters of each indicator occurrence. We chose this method, which has been applied in prior studies [17] because our interest was in identifying high geospatial concentrations of adverse perinatal health indicators that were unlikely to occur due to random chance. We compared the prevalence of the indicator in each census tract with that of neighboring tracts, identifying when a census tract exhibited a statistically significant spatial association with census tracts of higher prevalences, or hotspots, using an alpha cutoff level of 0.05 [18]. This technique can also identify significant geographic clusters of low values, also known as cold spots. We defined overlap as when a hotspot census tract was also designated as an area of environmental risk. To identify areas of increased adverse perinatal health risk, we counted the number of overlapping adverse perinatal health hotspots for each census tract from 0 (no adverse indicator hotspots) to 3 (census tract a hotspot for all three adverse indicators).

We then performed logistic regression analyses to assess associations between environmental risk areas and each adverse perinatal indicator, as well as between component scores and indicators. We also performed Poisson regression to assess associations between environmental risk areas and the number of overlapping hotspots. For regression analyses, we adjusted for parental birthplace (US-born/foreign-born) and parity (first live birth/not first live birth), as they are relevant to perinatal health [19, 20] and not integrated into the environmental risk area classification. As overall race and ethnicity within a census tract is incorporated into the environmental risk area designation (Table 1), we did not include race and ethnicity from the Bureau of Vital Statistics dataset. Lastly, we performed Spearman correlation analyses between the indicator rates to examine the strength of association among our three indicators.

Our final sample for analysis included 2101 census tracts (97% of total NYC census tracts). Starting with 2165 total census tracts, we excluded tracts that were non-residential (45 tracts) or had missing indicator data (16 tracts). We also excluded three tracts that had empty neighbor sets (i.e., tracts without neighboring tracts within five miles of their centroids) because Gi* statistic calculation requires neighboring tracts. The number of births represented for each of our indicators had no more than 1.6% missingness across the three indicators. All analyses were performed using R version 4.4.2 [21].

## Results

### Tract-Level Characteristics

Table 2 shows the descriptive characteristics of all census tracts included for analysis overall and by environmental risk area designation. Out of 2101 census tracts, 45.2% were classified as areas of environmental risk. Risk areas had higher proportions of pregnant residents who identify as Hispanic (*p* = 0.014) and non-Hispanic Black (*p* < 0.001) compared with non-risk areas. In non-risk areas, non-Hispanic White was the most reported race/ethnicity among birthing residents (44.4%), which differs from environmental risk areas where the most common race/ethnicity among pregnant residents was Hispanic (45.2%).

**Table 2:** Sample characteristics by environmental risk area designation

	All census tracts	Non-risk areas	Risk areas	*p*-value
Number of census tracts, %	2101	1152 (54.8%)	949 (45.2%)	
Non-adolescent birthing parent age, *mean prevalence (SD)*				
20–34	73.8% (9.55%)	72.4% (11.2%)	75.5% (6.58%)	0.23
35–44	21.6% (10.9%)	25.6% (11.8%)	16.7% (7.31%)	0.13
45+	0.0753% (3.92%)	0.124% (0.51%)	0.0161% (0.138%)	0.74
Birthing parent race/ethnicity, *mean prevalence (SD)*				
Hispanic	29.6% (24.4%)	16.7% (14.5%)	45.2% (24.9%)	0.014*
Asian/Pacific Islander	15.7% (19.7%)	19.9% (21.3%)	10.6% (16.1%)	0.34
Non-Hispanic White	30.4% (31.3%)	44.4% (32.0%)	13.5% (20.4%)	0.13
Non-Hispanic Black	24.3% (30.7%)	19.1% (32.5%)	30.6% (27.1%)	< 0.001*
Parental birthplace, *mean prevalence (SD)*				
US-born	47.4% (20.0%)	46.9% (20.3%)	48.0% (19.8%)	0.37
Foreign-born	52.6% (20.0%)	53.1% (20.3%)	52.0% (19.8%)	
Parity, *mean prevalence (SD)*				
First live birth	46.5% (9.96%)	48.5% (11%)	44.0% (7.79%)	0.24
Not first live birth	53.5% (9.96%)	51.5% (11%)	56% (7.79%)	
Preterm birth, *mean prevalence (SD)*	7.35% (3.8%)	6.49% (3.8%)	8.40% (3.54%)	0.068
Adolescent pregnancy, *mean prevalence (SD)*	4.49% (4.86%)	1.82% (2.92%)	7.73% (4.77%)	< 0.001*
Pre-pregnancy obesity, *mean prevalence (SD)*	16.9% (9.44%)	13.1% (9.02%)	21.5% (7.73%)	0.023*

*p-value < 0.05

### Hotspot Analysis

**Fig. 2:**
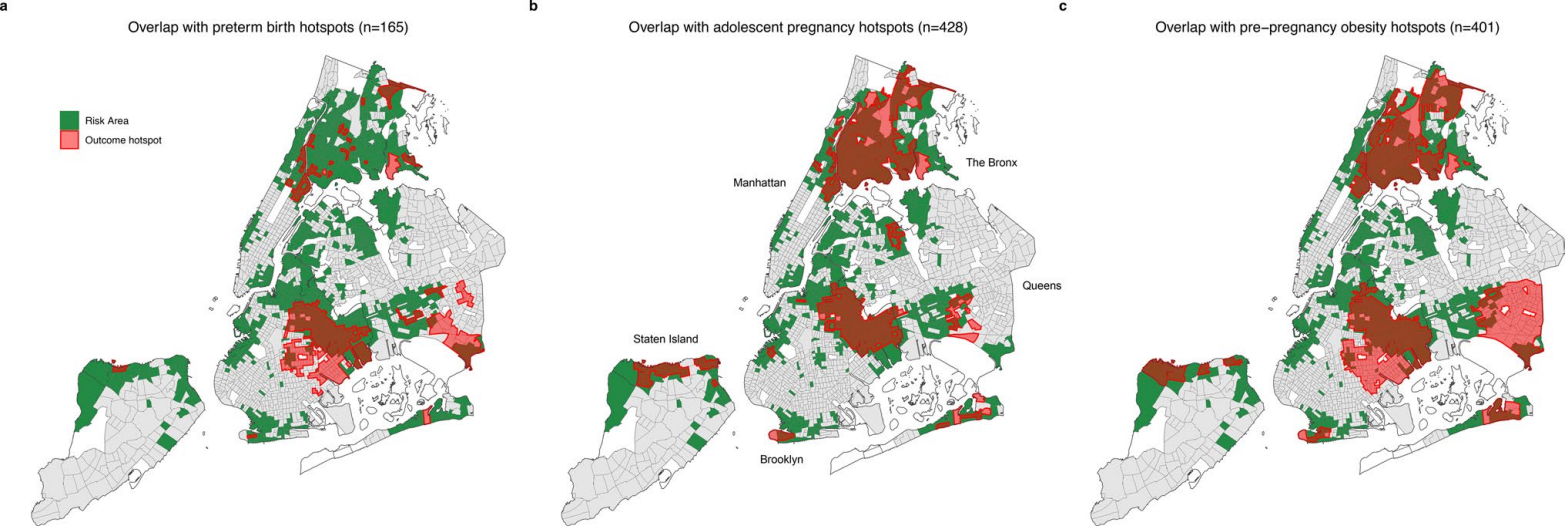
Overlap of environmental risk areas and hotspots of **a** preterm birth, **b** adolescent pregnancy, and **c** pre-pregnancy obesity

**Fig. 3:**
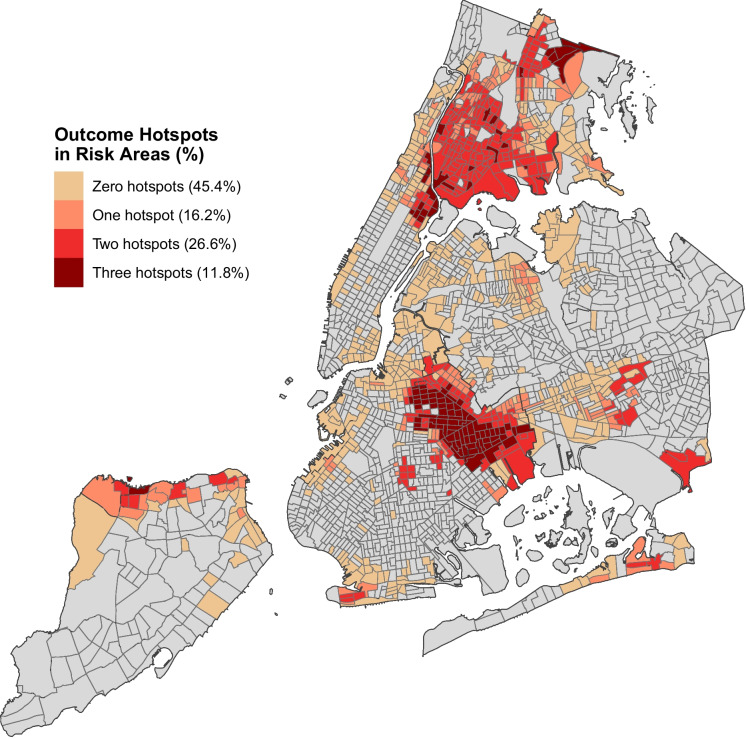
Perinatal indicator hotspot counts among environmental risk areas based on 2010 census tract boundaries

Figure 2 displays the overlap between environmental risk areas and the three adverse perinatal health indicators. The hotspot prevalence across census tracts for the indicators was as follows: 12.3% of all census tracts (*n* = 259) for preterm birth, 21.8% (*n* = 457) for adolescent pregnancy, and 28.4% (*n* = 596) for pre-pregnancy obesity. The proportion of environmental risk areas that were hotspots for all three outcomes was at least double the proportion of non-risk areas, with the rate of adolescent pregnancy hotspots being over 20 times as high in risk areas (Table 3). Figure 3 displays how the majority of environmental risk areas were hotspots for at least one indicator (54.6%). Overall, 726 (34.6%) census tracts were hotspots for at least one indicator, 467 (22.2%) were hotspots for at least two indicators, and 119 (5.7%) were hotspots for all three. Of the 119 census tracts that were hotspots for all three, 112 tracts (94.1%) were designated as areas of environmental risk. The two most closely correlated indicator hotspots across all census tracts were adolescent pregnancy and pre-pregnancy obesity, with a Spearman correlation coefficient of 0.559 showing a moderate correlation. Supplemental Figure 1 also shows the spatial distribution of perinatal health indicator cold spots, primarily across non-risk areas.

**Table 3:** Perinatal indicator hotspot distribution by environmental risk area designation

	All census tracts (*n* = 2101)	Non-risk areas (*n* = 1152)	Risk areas (*n* = 949)
Individual hotspots*, n (% of designation)*			
Preterm birth	259 (12.3%)	94 (8.2%)	165 (17.4%)
Adolescent pregnancy	457 (21.8%)	29 (2.5%)	428 (45.1%)
Pre-pregnancy obesity	596 (28.4%)	195 (16.9%)	401 (42.3%)
Number of overlapping hotspots*, n (% of designation)*			
0 hotspots	1375 (65.4%)	944 (81.9%)	431 (45.4%)
1 hotspot	259 (12.3%)	105 (9.1%)	154 (16.2%)
2 hotspots	348 (16.6%)	96 (8.3%)	252 (26.6%)
3 hotspots	119 (5.7%)	7 (0.61%)	112 (11.8%)

### Regression Analysis

Table 4 shows the results of our unadjusted and adjusted regression analyses. In both the crude and adjusted analyses, environmental risk area designation was associated with higher odds of a census tract being a hotspot for all perinatal health indicators. After adjustment, the odds of census tract hotspot status for risk areas versus non-risk areas were 2.11 for preterm birth (95% CI 1.60, 2.78), 32.8 for adolescent pregnancy (95% CI 21.8, 49.4), and 3.15 for pre-pregnancy obesity (95% CI 2.56, 3.87).

**Table 4:** Hotspots of perinatal indicator associated with environmental risk area designation

	Univariable			Multivariable		
Indicator hotspots	OR	95% CI	*p*-value	OR	95% CI	*p*-value
Preterm birth	2.37	(1.81, 3.10)	< 0.001*	2.11	(1.60, 2.78)	< 0.001*
Adolescent pregnancy	31.8	(21.5, 47.0)	< 0.001*	32.8	(21.8, 49.4)	< 0.001*
Pre-pregnancy obesity	3.59	(2.94, 4.39)	< 0.001*	3.15	(2.56, 3.87)	< 0.001*
**Overlapping hotspots**	**RR**	**95% CI**	***p*-value**	**RR**	**95% CI**	***p*-value**
	3.79	(3.34, 4.31)	< 0.001*	3.36	(2.96, 3.82)	< 0.001*

Multivariable models adjusted for parental birthplace and parity

*p-value < 0.05

Areas of environmental risk have 3.36 times the number of overlapping hotspots compared to non-risk areas, when adjusting for parental birthplace and parity (95% CI 2.96, 3.82). Looking at the component scores individually, we see that environmental burden score is only positively associated with hotspots of adolescent pregnancy (aOR = 1.03; 95% CI 1.01, 1.05), and the population characteristics and health vulnerability component score was associated with hotspots for all three indicators (Supplemental Table 1). This is consistent with the spatial trends shown in Supplemental Figure 2, where multiple hotspots tended to be in areas with greater population and health vulnerability scores.

## Discussion

### Main Findings

This ecological observational study examined relations between areas of environmental risk and hotspots of adverse perinatal health indicators in NYC. We found notable overlap in hotspots and areas of environmental risk in this urban setting: a majority of environmental risk areas were a hotspot for at least one adverse perinatal health indicator. We also found that environmental risk area designation was associated with hotspots of preterm birth, adolescent pregnancy, and multiple adverse indicators. These findings reflect our hypothesis, highlighting the interconnected nature of environmental threats and perinatal health. With worsening environmental risks [22], our results support the creation of policies directed at identifying high-risk environmental areas, given their demonstrated association with significant perinatal health indicators. More than half of all environmental risk areas were hotspots for at least one indicator, and, among these, most were hotspots for multiple indicators as seen in parts of the Bronx, Brooklyn, and northern Manhattan. These same areas are known to have higher relative economic inequalities and health disparities [23, 24]. The Poisson regression shows that identified areas of environmental risk were associated with 3.36 times the number of adverse perinatal health indicator hotspots. Stratified analysis based on component scores shows that these associations tend to be more driven by population characteristics and health vulnerabilities which may have collinearity with the health indicators considered in this study. Environmental and climate burden component score was positively associated with hotspots of adolescent pregnancy, providing insight into how neighborhood disinvestment may have wide-ranging effects on population health. These insights can inform future strategies to address high rates of adverse perinatal health indicators in communities related to structural disparities of environmental risk, which are more concentrated in urban cities like NYC.

We identified significant overlap and associations between hotspots of preterm births and environmental risk area designations. A majority of preterm birth hotspots were environmental risk areas, totaling 165 census tracts out of 259 classified as hotspots (63.7%). Previously conducted geographic studies have found greater frequency of preterm birth in communities that have been historically redlined, experience greater poverty, and have higher concentrations of Black and Hispanic communities [7, 25]. Individual environmental hazards (e.g., extreme heat, water contaminants, and air pollution) have been associated with an increased risk of preterm birth [4, 26], and our findings support that the cumulative influence of multiple environmental threats may be associated with higher rates of preterm birth on the population level.

We also found that 428 of the 457 adolescent pregnancy hotspot census tracts were areas of environmental risk (93.7%). Unlike preterm birth, there is no direct pathway between environmental exposures and rates of adolescent pregnancy elucidated in the literature. Thus, as hypothesized, while environmental risk areas were associated with adolescent pregnancy hotspots, the distribution of adolescent pregnancy hotspots differed from the hotspots of preterm birth. However, this was the only perinatal health indicator that showed an association with census tract environmental and climate burden component scores. Adolescent pregnancies in New York City show disparities by race/ethnicity and neighborhood disinvestment, aligned with prior literature demonstrating associations between adolescent pregnancy and higher social risk [27]. The overlaps and associations we detected may stem from the greater prevalence of adolescent pregnancies in communities with persistent poverty, where logistical and societal barriers to reproductive healthcare access are common [28]. Even with the use of socioeconomic composite indices, ecological studies have provided evidence that areas experiencing disadvantage face higher rates of adolescent pregnancy [29, 30]. These same communities often face disproportionate exposure to environmental threats, which our findings suggest may be associated with community risk for adverse perinatal outcomes.

We detected overlaps between areas of environmental risk and hotspots of pre-pregnancy obesity. Pre-pregnancy obesity—associated with preterm birth, congenital defects in offspring, and childhood obesity risk [15]—is the most prevalent of our three indicators. According to data from 2021, 31.3% of pregnant residents in New York State were classified as obese prior to pregnancy [31]. Among the 596 census tracts within hotspots of pre-pregnancy obesity, 401 of those census tracts were environmental risk areas (67.3%), aligning with prior evidence finding that obesity in the general population is associated with neighborhood food environments, walkability, and other socioeconomic factors [32, 33]. Notably, one study highlighted that this association was confounded by the percentage of Black residents in the neighborhood [32]. Specifically for pre-pregnancy obesity, an ecological study on metropolitan cities in the USA similarly found that an index of 11 indicators of area deprivation, among other city-level factors, was a significant risk factor for pre-pregnancy obesity prevalence [34]. Communities with higher exposure to environmental hazards may face increased risks of obesity additionally due to increased contact with endocrine-disrupting chemicals, which interfere with hormonal systems that regulate metabolism, fat storage, and energy balance [35].

### Strengths and Limitations

The strengths of this study include the high-quality data from the Bureau of Vital Statistics, a large sample size of births, and the use of the comprehensive environmental risk area designation. Previous studies have examined the relation between various environmental hazards and climate-related events, but few have looked at the use of a composite of these exposures with multiple perinatal health indicators. This is crucial since environmental hazards are often simultaneously present. A similar metric, the former federal Climate and Economic Justice Screening Tool, assesses comparable burdens and has served as guidance for state, territory, and tribal governments in their approaches to environmental justice screening, last updated to have 35 factors in August 2024 [9]. The National Academies of Sciences, Engineering, and Medicine has called for validated geospatial and cumulative tools for fair environmental health, and our study provides some validity for this policy designation system in the context of perinatal health burden [9, 36]. The ecological nature of this study and scope of the data used are strengths for the surveillance of pregnant populations in NYC.

Due to the ecological nature of our study, however, our findings neither determine causality nor draw individual-level conclusions (i.e., assessing one’s individual risk of adverse indicators from living in an environmental risk area). Some initially considered environmental risk indicators that would have provided valuable information on community burden were dropped due to lack of usable data [16]. Additionally, six of the 45 environmental indicators were collected after perinatal health outcome assessment in 2020. These variables were not expected to have changed substantially as they were based on pre-existing neighborhood infrastructure. Evidence has shown that the validity of maternal weight data reported on vital records may be poor due to recall bias [37]. This health indicator was still chosen due to the previously identified relationship between environmental factors and body mass index. Lastly, the inclusion of data before 2020 may not be reflective of the current environment following the COVID-19 pandemic. This temporal adjustment calls for a reevaluation of this association and across other health domains once updated data are released.

### Interpretations

As clinical and community-based shareholders develop strategies to connect individuals at risk for adverse perinatal outcomes to services, our findings contribute to evidence that integrating knowledge of residence in environmental risk areas could help guide efforts to mitigate exposures and deliver more targeted care [38]. The New York 1115 Medicaid Waiver amendment, running from 2024 to 2027, aims to improve health quality and outcomes in areas with historical health discrepancies and disengagement [39]. Environmental risk area classifications could help inform this goal by enabling clinicians to integrate geospatial data into screening efforts.

Structural conditions leading to heightened environmental threats have resulted in communities being at a greater risk of adverse exposures and subsequent disease burden [40, 41]. For leaders and policymakers, principles of environmental health and fairness should be considered when approaching health promotion, as infrastructural and societal barriers perpetuate environmental risk and burden. As expected, given that race and ethnicity frequently overlaps with social and economic disadvantage, we detected differences by environmental risk area designation in the proportion of pregnant residents who identified as Hispanic or Black. Previous studies have repeatedly identified disparities in environmental risk for predominantly Black and Hispanic neighborhoods in NYC [42, 43]. The use of environmental risk areas offers a more targeted and actionable basis for resource allocation and intervention and shifts the focus from individual characteristics and behaviors as drivers of perinatal risk to environmental conditions shaped by modifiable policies. This study represents a framework by which policy-derived designations can be tested for their relationships with health indicators to foster transparency, integrity, and social engagement.

## Supplementary Information

Below is the link to the electronic supplementary material.

ESM1 (PDF 4.92 MB)

ESM2 (PDF 11.2 MB)

ESM3 (DOCX 17.0 KB)

## Data Availability

Environmental risk areas were identified by the NYC Mayor's Office of Climate & Environmental Justice according to New York State's Disadvantaged Communities designation. Complete information on designation and scoring can be found on New York's Open Data Portal (https://data.ny.gov/Energy-Environment/Final-Disadvantaged-Communities-DAC-2023/2e6c-s6fp/about_data). Perinatal Health count data is available through the NYC Department of Health and Mental Hygeine's Public Use Birth Datasets (https://www.nyc.gov/site/doh/data/data-sets/public-use-birth-datasets.page).
