# Supplementary figures and images for: Environmental Risk and Adverse Perinatal Health Indicators in New York City: A Geospatial Hotspot Analysis
Source: J Urban Health. 2026 Mar 20;103(2):244–55. doi: 10.1007/s11524-026-01060-8 (PMC13235679; doi:10.1007/s11524-026-01060-8)

**a**

Preterm birth clusters

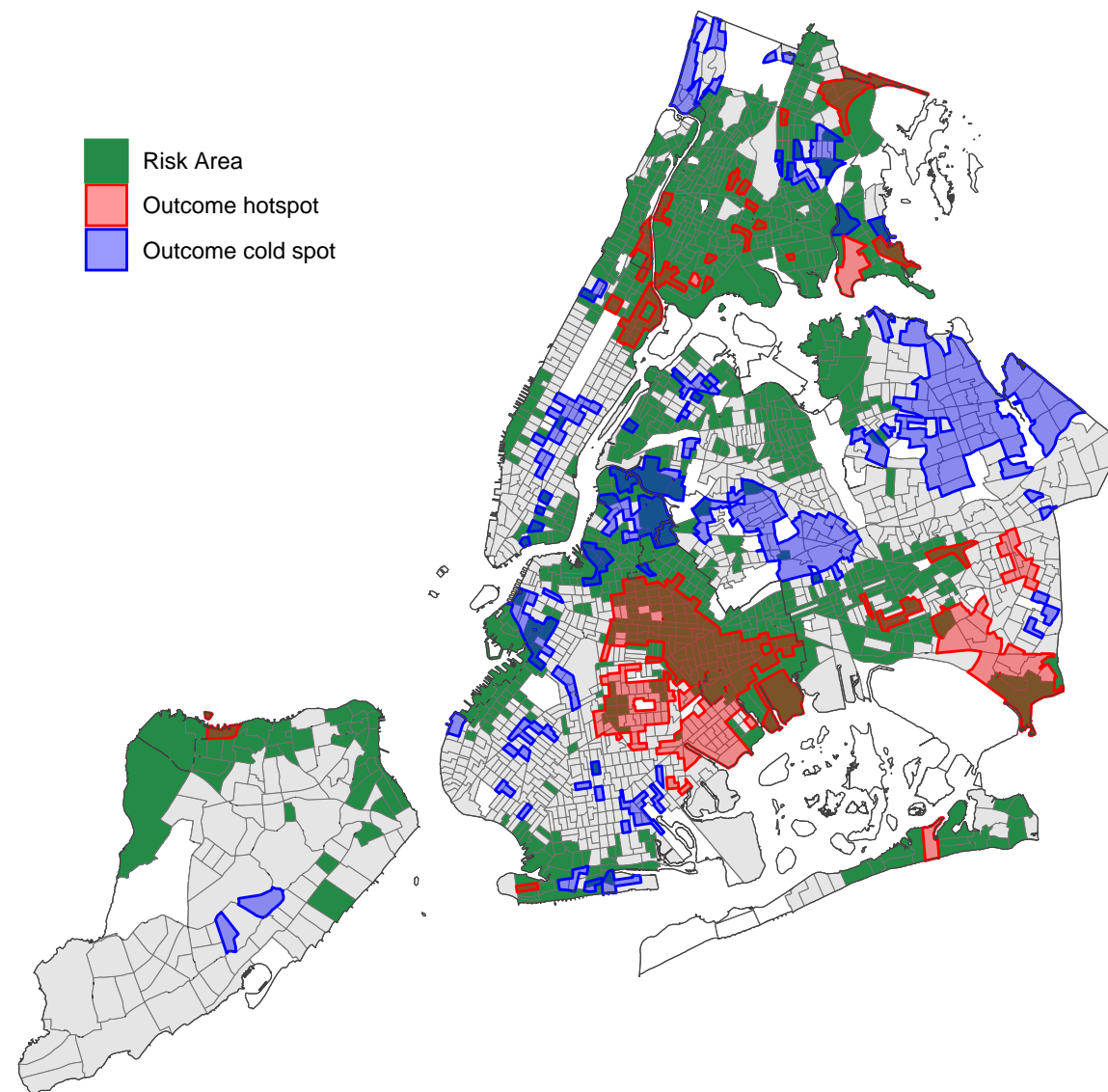**b**

Adolescent pregnancy clusters

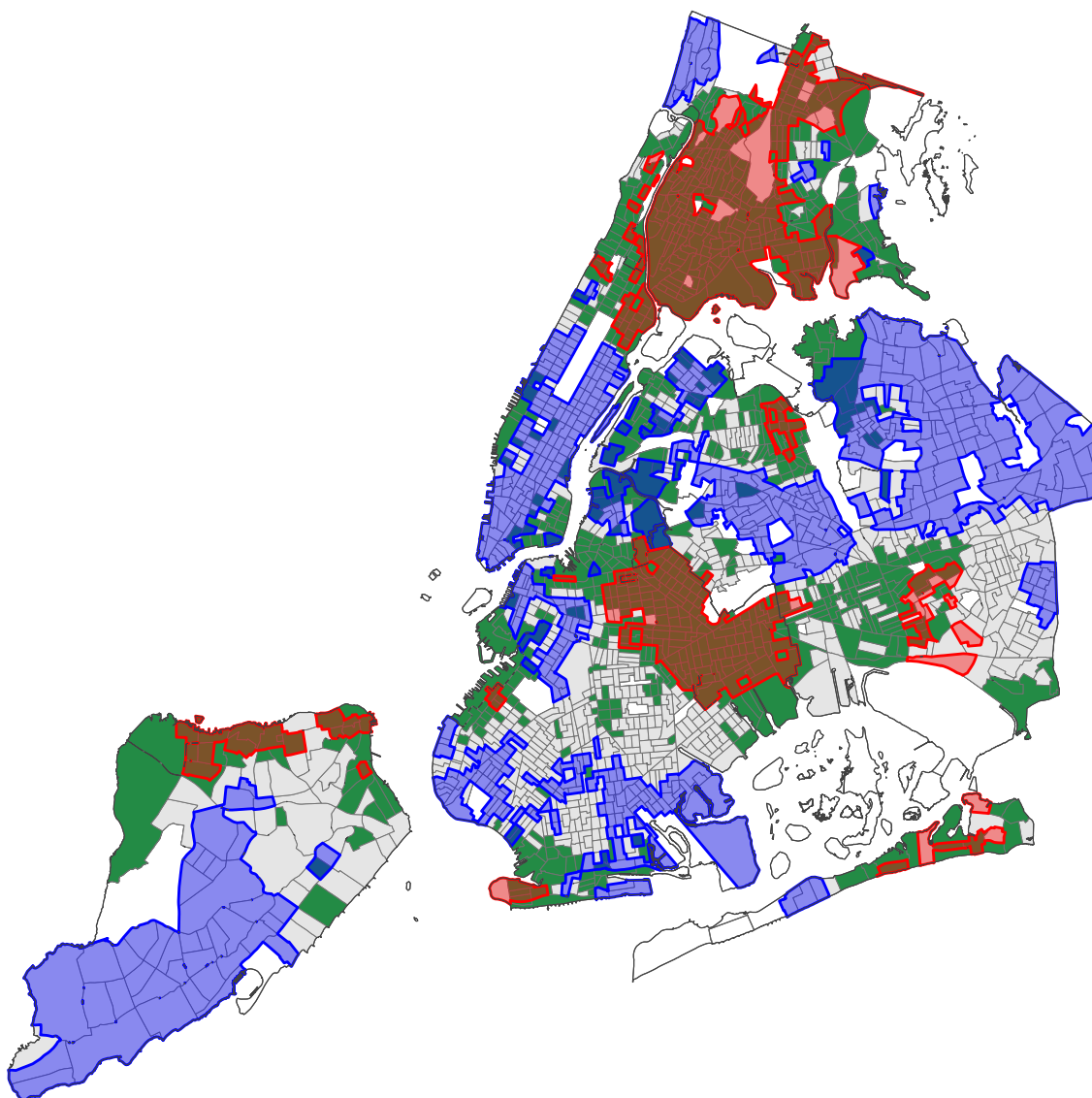**c**

Pre-pregnancy obesity clusters

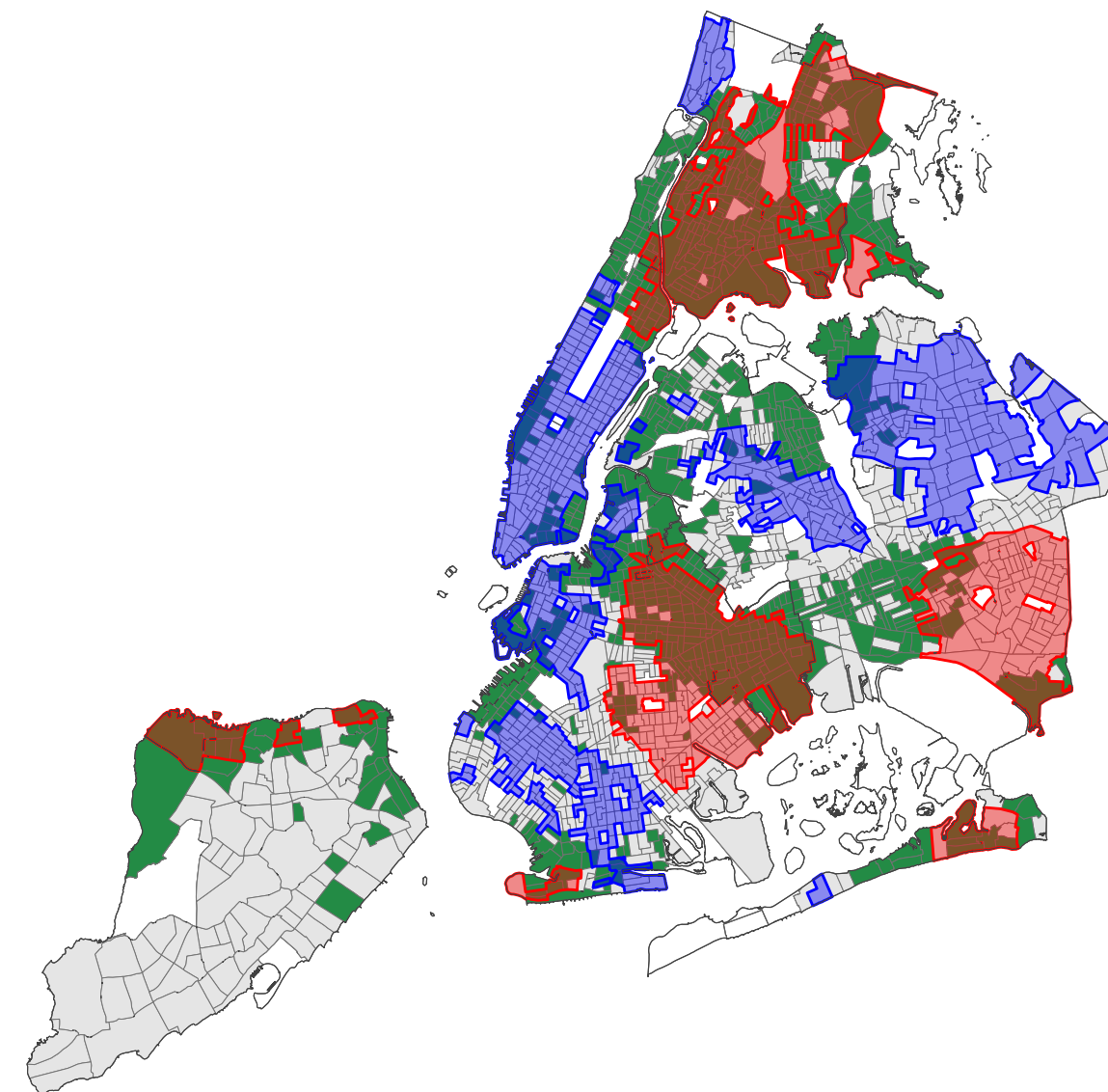

Supplement: Supplementary file 1 — (PDF 4.92 MB) [file 11524_2026_1060_MOESM1_ESM.pdf]

**a**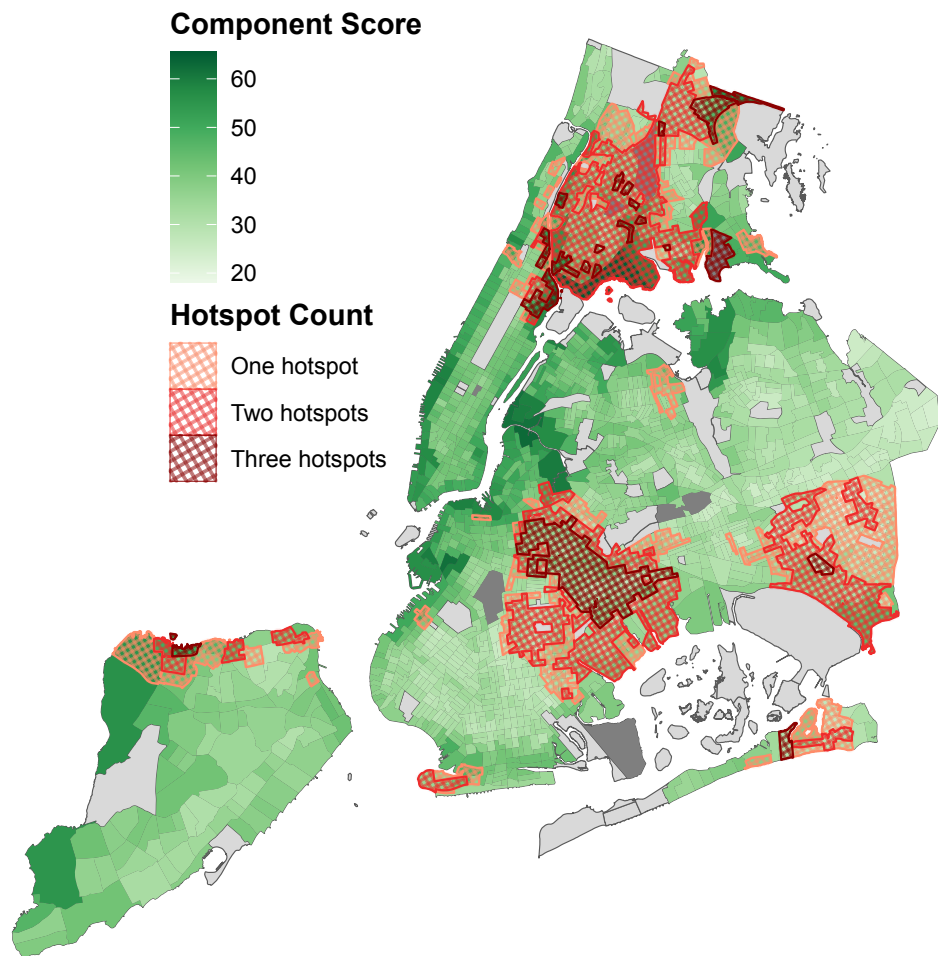**b**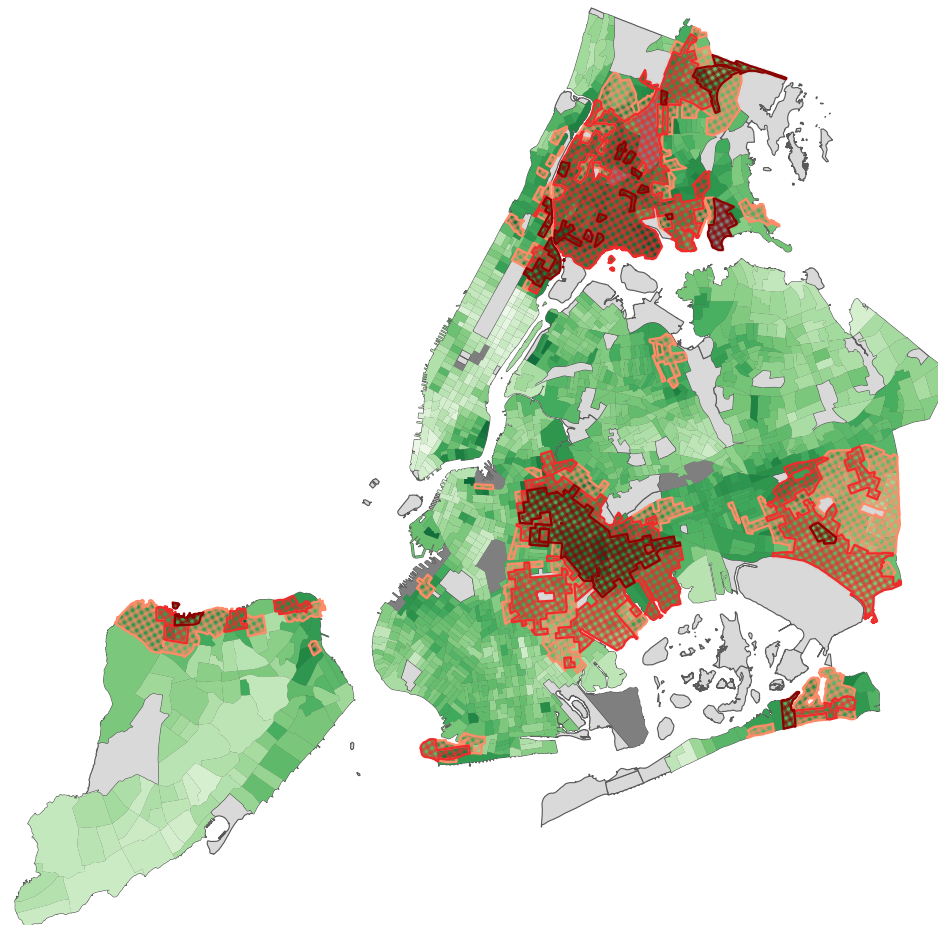

Supplement: Supplementary file 2 — (PDF 11.2 MB) [file 11524_2026_1060_MOESM2_ESM.pdf]
