# Supplementary material for: Environmental Risk and Adverse Perinatal Health Indicators in New York City: A Geospatial Hotspot Analysis
Source: J Urban Health. 2026 Mar 20;103(2):244–55. doi: 10.1007/s11524-026-01060-8 (PMC13235679; doi:10.1007/s11524-026-01060-8)
Supplement: Supplementary file 3 — (DOCX 17.0 KB) [file 11524_2026_1060_MOESM3_ESM.docx]

1. Supplemental Table 1: Hotspots of perinatal indicator associated with environmental risk component scores

|  | **Environmental burden and climate change risks** | | | **Population characteristics and health vulnerabilities** | | |
| --- | --- | --- | --- | --- | --- | --- |
| **Indicator hotspots** | **OR** | **95% CI** | **p-value** | **OR** | **95% CI** | **p-value** |
| Preterm birth | 0.95 | (0.92, 0.97) | <0.001* | 1.05 | (1.04, 1.06) | <0.001* |
| Adolescent pregnancy | 1.03 | (1.01, 1.05) | <0.001* | 1.24 | (1.21, 1.27) | <0.001* |
| Pre-pregnancy obesity | 0.95 | (0.93, 0.96) | <0.001* | 1.1 | (1.09, 1.11) | <0.001* |
| *p-value < 0.05 | | | | | | |
| Adjusted for parental birthplace and parity | | | | | | |
